# Prevalence and Factors Associated with Mental Health Complaints in Japanese Collegiate Athletes

**DOI:** 10.3390/sports12090240

**Published:** 2024-09-02

**Authors:** Takeshi Kimura, Aleksandra Mącznik, Akira Kinoda, Yuichi Yamada, Yuki Muramoto, Yoshinori Katsumata, Kazuki Sato

**Affiliations:** Institute for Integrated Sports Medicine, School of Medicine, Keio University, 35 Shinanomachi Shinjuku-ku, Tokyo 160-8582, Japan; takeshikimura4@gmail.com (T.K.); alexmacznik@gmail.com (A.M.); akirakiak@keio.jp (A.K.);

**Keywords:** mental health, collegiate athlete, surveillance, epidemiology

## Abstract

Investigations into mental health epidemiology in various cultural contexts were one of the main recommendations by the recent consensus statement on mental health in athletes, but the evidence in different sporting populations is rising slowly. This study aimed to investigate the prevalence of mental health complaints in Japanese collegiate athletes. The online survey was sent to the Japanese collegiate athletes associated with UNIVAS asking about the mental health complaints experienced during their sporting careers. Out of 10,999 athletes, 269 (2.4%) reported at least one complaint. Mental health complaints were more prevalent in female (3.6%) than male (1.5%) athletes. Skill sports had the highest prevalence (4.1%) of mental health complaints, and power sports had the lowest prevalence (1.5%) of mental health complaints. This study found a very low prevalence of self-reported mental health complaints in Japanese collegiate athletes. Preventive efforts should focus on monitoring early symptoms (rather than diagnoses), especially in skill sports and female athletes. Anxiety (38%) and depression (35%) were the most reported complaints and should be targeted first.

## 1. Introduction

Athletes are exposed to a tremendous number of stressors that can impact their health on many levels [1]. Training of the athletes is demanding, both physically and mentally, as it focuses on a constant push towards the edges of what is possible. Harsh qualification procedures, constant striving for perfection, and continuous risk of sustaining an injury are contributing to high-demand cultures in sports. Athletes are exposed to strict regimes in their diet, sleep, recovery, and training schedules that require sacrifices in other areas of life. Reduced opportunities for social events may lead to loneliness and difficulties in building close relationships and support networks. Traveling to competitions and, sometimes, even staying in residential sports facilities for prolonged periods deprive athletes of contact with their families. Moreover, the best competitive years overlap with the onset of many mental health disorders, which could put athletes at higher risk than the general population [2,3]. These specific factors characterizing the reality of being an athlete may contribute to the heightened prevalence of mental health complaints in the athletic population and, therefore, need separate investigation.

Indeed, mental health symptoms and disorders (defined as those involving distress, anxiety, depression, or substance misuse among others) have been reported previously to affect approximately 30% of athletes at some point in their sporting career [4,5]. These data, however, are limited to mostly elite athletes [6] and vary considerably between nations, sports, and sexes [7]. For example, at least one mental health problem was found in 46% of elite Australian athletes [8], while depression has been reported in 15% of German elite athletes [9]. Eating disorders were present in 18% of female elite Norwegian athletes, but if only leanness sports were considered, this number went up to 25% [10]. Investigations into mental health epidemiology in various cultural contexts were one of the main recommendations by the recent consensus statement on mental health in athletes [11]. Still, the evidence in different sporting populations is rising slowly.

The data on mental health in the Japanese athletic population have just started emerging but mainly at the professional team sports level and in male athletes [12,13,14]. Mental health is still a taboo topic in Japanese culture and, therefore, reporting is scarce [13], which makes any treatment efforts hard and preventive endeavors close to impossible. Similarly, comparable investigations are needed in countries internationally to clarify which aspects of mental health are universal globally and which are dependent on the cultural context. Therefore, the need for a broader investigation is warranted.

Mental health is of paramount importance to athletes, both for immediate performance and long-term well-being. Athletes at the elite level have been shown to be more psychologically healthy than their less successful counterparts [15]. Furthermore, the consequences of unrecognized and untreated mental health issues can lead to suicidal ideation and, therefore, are extremely serious. Sensible investigations into the prevalence of mental health complaints are immediately needed to guide the efforts of prevention, early diagnosis, and effective management of mental health complaints in athletes [6].

This study aimed to investigate the prevalence of mental health symptoms in Japanese collegiate athletes, both males and females. Additionally, the factors associated with reporting mental health complaints were analyzed, as were the differences between females and males and the differences between different sport groups.

## 2. Materials and Methods

This was a retrospective, cross-sectional, observational study approved by the Ethics Committee of Keio University (approval number: 20211158) and run in accordance with the principles of the Declaration of Helsinki [16]. The design and reporting of this study are based on the STROBE-SIIS consensus statement [17].

Japanese collegiate athletes associated with UNIVAS (Japan Association for University Athletics and Sport) were eligible and invited to take part in this study. UNIVAS affiliates approximately 170,000 collegiate athletes (127,000 males, 43,000 females), and simple random sampling was applied to recruit the athletes through letters and phone calls to universities and sporting organizations. After the athletes read the information sheet and agreed to participate, they were invited to take an online survey.

This survey was published on a purposefully built website (https://enquete.cc/q/BC2XC8A8 (accessed on 1 June 2022)) and collected data from June 2022 to August 2023. The questions were adapted from the Japanese Society of Clinical Sports Medicine and the Japanese Society for Athletic Training consensus document [18] to best suit the collegiate population. The questions were pilot tested and face and content validated using expert sports medicine and psychiatry physicians [19]. In brief, the survey asked about athlete characteristics (age, sex, weight, height) and their sports participation (sport played, experience, frequency of training, the highest level of participation in the past—junior high and high school). Additionally, the athletes were asked about any mental health diagnosis they might have received during their sporting career (none, anxiety, depression, sleep disorders, eating disorders, developmental disorders, alcohol misuse, substance misuse). Anxiety was defined as “excessive worry that is difficult to control, cause significant distress and impairment, and occur on more days than not for at least six months” [20]. Depression was defined as “feelings of sadness, despair, emptiness, discouragement, or hopelessness; having no feelings; or appearing tearful” [19]. Sleep disorders included symptoms like insomnia, sleep-related breathing disorders, circadian rhythm sleep–wake disorders, or sleep-related movement disorders [21]. Eating disorders were “characterized by a persistent disturbance of eating behavior that impairs health or psychosocial functioning” [22]. Developmental disorders were defined as disorders originating in childhood that involve serious impairment in different areas, such as developmental language disorder, learning disorders, motor disorders, and autism spectrum disorders, also including ADHD [23]. Alcohol and substance misuse was defined as use that is difficult to control or causes distress or impairment in social, occupational, or health contexts [24].

For analysis, the athletes from different sports were grouped into 4 groups. The group division was performed on the basis of similarities in the relative isometric and isotonic components of the sport and their potential influence on cardiovascular adaptations and risks, including sudden death [25,26,27]: (1) skill sports (only mild cardiovascular demand), (2) power sports (short bursts of intense exercise but short cumulative duration), (3) mixed sports (alternating phases of exercise and recovery), and (4) endurance sports (high dynamic and static components). Additionally, as the Japanese culture strongly involves aesthetics, we have separated sports with aesthetic components to form group (5), aesthetic sports. (The specific sports included in each group with sample size and number of females and males are presented in Table 2).

The statistical analysis was performed using SPSS Statistics for Macintosh (IBM Corp, Armonk, NY, USA. Released 2021 version 28.0). Continuous data were summarized with mean and standard deviation, and categorical data were summarized with frequencies and percentages. Normality (Shapiro–Wilk) and homogeneity of variance (Levene’s test) within the data were tested. The prevalence of mental health complaints was calculated by dividing the number of athletes who reported a complaint by the number of all athletes and multiplied by 100%. Differences in the proportions of complaints between females and males were compared using the chi-square test. Differences in athlete characteristics between groups of athletes who reported mental health complaints and those who did not were compared with Mann–Whitney (all variables violated the normality assumption, and all but ‘practice days per week’ violated the homogeneity assumption) or chi-square tests, depending on the type of the variable. A *p*-value of <0.05 was considered statistically significant.

## 3. Results

We received data from 11,000 Japanese collegiate athletes (one of the athletes was excluded with an incorrect survey answer), 2.4% of whom reported at least one mental health complaint. The athletes’ characteristics are presented in Table 1. The athletes who reported mental complaints were shorter, weighed less, had lower body mass index (BMI), had longer sports experience, had fewer practice days per week, and more frequently reported illness than the athletes who did not report any mental health complaints.

The sports represented in this study are summarized in Table 2. The most represented group of sports was mixed sports (77.6%), and the least represented was aesthetic sports (1.8%).

In Table 3, we present the prevalence of mental health complaints reported within each sports group. The total prevalence of mental health disorders differed significantly between the sports groups. Additionally, differences in the frequency of reporting between different sports groups were found for anxiety, sleep symptoms, and alcohol misuse.

Table 4 depicts the prevalence of mental complaints in females and males. Females reported a higher prevalence of total mental health complaints but also a higher prevalence of anxiety, depression, sleep disorders, and eating disorders than males.

The characteristics of the female athletes are reported in Table 5. The female athletes who reported mental complaints were lighter, had lower BMI and sports experience, and trained fewer days per week on average than the ones who did not report mental complaints.

The characteristics of the male athletes are presented in Table 6. The male athletes who reported mental complaints had significantly fewer practice days per week than the athletes who did not report any mental health complaints.

In Table 7, we present the significant differences in athlete characteristics for each mental health complaint. Height and weight differences were found for anxiety, depression, and eating disorders, but BMI differences were only found for eating disorders. Sports experience differences were found for depression. Differences in practice days per week were found for anxiety, depression, sleep disorders, developmental disorders, and substance misuse.

The athletes who reported anxiety were, on average, 3.2 cm shorter in height and 4.5 kg lighter in weight and practiced half a day less per week. The athletes who reported depression were, on average, 2.9 cm shorter and 4.3 kg lighter, had 1 year less of experience, and practiced their sport 0.3 days less per week. The athletes who reported sleep disorders were, on average, training 0.3 days per week less than the athletes who did not report sleep disorders. The athletes who reported eating disorders were, on average, 5.1 cm shorter and 8.4 kg lighter and had 1.7 lower BMI. Developmental disorders were reported by the athletes who, on average, practiced their sport 0.6 days less per week. The athletes who reported substance misuse reported one and a half fewer practice days per week. No differences were found in athletes’ characteristics for alcohol misuse.

## 4. Discussion

This was an investigation into mental health complaints in a large sample of Japanese athletes. Experiencing mental health complaints at some point in their sporting career was reported by 2.4% of 10,999 Japanese collegiate athletes, with anxiety and depression being the most common complaints. More female athletes (3.6%) than male athletes (1.7%) reported mental health complaints. The highest prevalence was reported in skill sports (4.1%), and the lowest was reported in power sports (1.5%). The athletes with smaller bodies, shorter sports experience, and less frequent training were more likely to report mental health complaints.

### 4.1. Total Prevalence of Mental Health Complaints

The 2.4% prevalence of mental health complaints found in this sample of collegiate Japanese athletes is extremely low when compared with the data collected in elite Japanese athletes (3.1% prevalence of mental illness in the past in professional soccer players or 23–53% of complaints in professional rugby players) but also internationally (19–34% in current elite athletes and 16–26% for former elite athletes) [4,10,12,14,28]. This discrepancy is probably due to the varying methods of data collection used between the studies. In our study, a simple question ‘Which of the following mental health complaints were diagnosed with during your sporting career’ was asked. This allowed for the athletes to reflect on their mental health and answer which complaint they were diagnosed with. In other studies, various measurement tools were used to gather and assess the number and magnitude of symptoms and subsequently diagnose (or not) specific mental complaints. This suggests that it may take a long time or significant worsening of symptoms before an athlete recognizes they have a mental health issue and seeks help from a health professional.

We suggest that various symptoms could be present before the athlete can identify and formulate a mental health complaint. This phenomenon was previously highlighted in a similar population where, in a longitudinal survey, the reporting of vulnerability proceeded and predicted depressive symptoms [29]. Additionally, previous studies showed that athletes are reluctant to seek mental health help due to the sporting environment generally appraising toughness and tenacity and despising any signs of weakness, be it physical or mental [1,13]. Therefore, implementing an ongoing monitoring of mental health symptoms and implementing processes for managing these symptoms before they escalate or worsen could be useful in the long-term prevention and management of athletes’ mental health.

### 4.2. Prevalence of Specific Mental Health Complaints

#### 4.2.1. Anxiety and Depression

Anxiety (38%) and depression (35%) were reported as the most prevalent of all mental health complaints by the Japanese collegiate athletes in this study. The absolute numbers of these complaints were very low though, with 1% and 0.9% of athletes reporting anxiety and depression, respectively. These numbers are extremely low when compared to the data presented in the literature. Thirty-two percent (81/251 players) of Japanese professional rugby players reported mild, 5% (12/251 players) reported moderate, and 5% (13/251 players) reported severe anxiety and depressive symptoms within the last 30 days [14], and another study found the prevalence of depressive symptoms in Japanese professional soccer players at 9.4% (62/661 players; in the past two weeks) [12]. Additionally, 14% (5/36) of British national squad swimmers met a threshold for major depressive episodes in the last 2 weeks [10]. These numbers matter as depression may lead to suicidal ideation reported in athletes at 6–8% in previous studies, a serious life-threatening event [14,30]. Therefore, monitoring and ongoing support are of paramount importance in this population.

#### 4.2.2. Sleep Disorders

Sleep disorders were reported by 0.7% of the athletes in this study. Again, a very low number when compared to the literature, where disturbed sleep was identified in 11% of British national squad swimmers [30] and poor sleep was reported by 54% of NCAA soccer players [Benjamin]. Sleep duration and quality are of paramount importance for athletes, as inefficient sleep was associated with both lower performance and a higher risk of sustaining sport-related injuries [31,32,33,34,35].

#### 4.2.3. Eating Disorders

The prevalence of eating disorders reported by the athletes in this study was 0.3%. The only study that reported a lower prevalence was the study that reported 0% of adverse nutrition behaviors in a sample of 36 British national squad swimmers [30]. In another study, as many as 60% of Norwegian elite female athletes were classified as at risk of the female athlete triad (disordered eating, amenorrhea, and osteoporosis), which was lower in comparison to non-athlete controls (69%) [10]. This difference grew to 59% versus 74% for the 20–29-year-old age group [10]. Fifteen percent of athletes had an eating disorder and body dissatisfaction in Norwegian elite female athletes, which was more than non-athletes (28%) [10]. Body dissatisfaction was previously associated with a higher risk for eating disorders [36].

#### 4.2.4. Developmental Disorders

Developmental disorders (e.g., developmental language disorder, learning disorders, motor disorders, and autism spectrum disorders, also including ADHD—attention deficit hyperactivity disorder) were reported by 0.2% of the athletes in this study. A recent review of 17 studies reported the prevalence of ADHD in athletes varied between 4.2% and 8.1% [37] and was associated with advantages for athletes, such as rapid reaction and switching attention to emerging stimuli [38]. Minor neurological dysfunction was detected in 18% of male youth athletes from three European countries, leading predominantly to deficits in the fine motor domain [39]. Developmental disorders are probably the hardest to self-diagnose from all the mental health complaints and, therefore, expected to be low in this study.

#### 4.2.5. Alcohol and Substance Misuse

Alcohol misuse was reported by 0.04%, and substance misuse was reported by 0.03% of the athletes in this study, which is low. In general, athletes report lower rates of alcohol use than the general population, but binge drinking, specifically, has been reported with high prevalence in American elite-level sports (both females and males) [40] and prospective Division I athletes [41]. However, adverse alcohol use was reported as high as 22.9% in British national team swimmers [10]. Sixty-eight percent (n = 490) of American collegiate softball players drank alcohol on at least one occasion in the previous 30 days, and 51% (n = 368) engaged in heavy episodic drinking on at least one occasion in the previous 30 days [42]. Alcohol use has been previously related to psychological distress and stress in American collegiate athletes [41].

General drinking motives and sport-related drinking motives in conjunction were suggested as motives for athlete alcohol use in collegiate softball players; however, the same study found that the athletes who reported individualistic reasons for drinking (rather than social or team-related reasons) had higher alcohol consumption and higher consequences [42]. Australian Football League players showed distinct drinking patterns related to the time of the year, with low risky consumption for most of the season (2%) and high risky consumption at the end of the season (54%) and during vacations (41%) [43]. Either way, the sporting environment can and will influence athlete use of alcohol and, therefore, should be considered in prevention efforts.

The low prevalence of reporting of alcohol misuse found in this study may be attributed to the fact that alcohol consumption by adults in Japan is socially accepted and part of the work culture. Therefore, although misuse was rarely reported in this study, the overall consumption may still be high. Cocaine, cannabis, opioids, and tobacco use were suggested at 2–5% prevalence in the athletic population [44]. The low prevalence of substance misuse found in this study is interesting; although some substances (like narcotics) are illegal and severely punished in Japan, others like tobacco are widely used. Investigations into substance use in the athletic population are scarce and needed, as the consequences of misuse are severe and their impact goes well beyond sports.

### 4.3. Mental Health Complaints in Female versus Male Athletes

More female athletes (3.6%) than male athletes (1.7%) reported mental health complaints. Females reported double the prevalence of anxiety, depression, and sleep disorders compared to males. The prevalence of eating problems showed the biggest difference, with females reporting 84% of all eating problems in this study. This agrees with the findings of studies on elite athletes in Australia and Norway and high-performance collegiate athletes in Iran and France that also reported a higher prevalence of mental health complaints in females [5,28,45,46]. The total prevalence of mental health complaints was higher, with 27% of Australian female athletes admitting to ever receiving psychological treatment [5], 20% of elite Norwegian female athletes being classified as at risk of an eating disorder [46], 20.2% of high-level French female athletes having at least one psychopathology [28], and 31% of high-performance collegiate female athletes considered having depressive symptoms [45].

More factors associated with mental complaints were found for female athletes than for their male counterparts. The female athletes who reported mental health complaints had, on average, lower weight and BMI, trained 0.3 days less per week, and had 0.8 years less experience in their sport than the females who did not report any mental health complaints. The male athletes who reported mental health complaints practiced half a day less per week than the males who did not report mental health complaints.

### 4.4. Mental Complaints in Different Sports Groups

We found a difference in the prevalence of mental complaints between different sports groups. The athletes performing skill sports (e.g., aviation, table tennis) reported the highest total prevalence (4.1%) of mental health complaints, whereas the athletes from power sports (e.g., judo, wrestling) reported the lowest (1.5%) total prevalence. It could be related to the skill sports requiring considerable involvement of the nervous system, whereas power sports balance the requirements between the nervous system and physical capacities.

Differences between sports groups were also found for specific mental health complaints. In this study, anxiety and sleep problems had the highest prevalence in skill sports. Alcohol misuse was reported most often in aesthetic sports.

On the contrary, differences between sports groups were not found in the prevalence of depression, eating problems, developmental problems, and substance misuse. In the literature, a higher percentage of Norwegian female elite athletes competing in aesthetic sports were classified as at risk of the female athlete triad compared with athletes competing in ball game sports [10]. Also, a higher percentage of athletes competing in aesthetic sports were underweight compared with athletes competing in technical, ball game, and power sports [10]. A higher percentage of athletes competing in technical sports than athletes competing in endurance sports scored higher on the eating disorder and body dissatisfaction scale [10].

In Norwegian elite female athletes, a significantly higher proportion of athletes in aesthetic sports and weight-dependent sports than in ball games and power sports were classified as at risk of developing eating disorders [46]. Significantly fewer athletes in endurance sports than aesthetic and power sports were classified as at risk of developing an eating disorder [46].

### 4.5. Athlete’s Characteristics Associated with Mental Health Complaints

In general, this study found that athletes who reported mental complaints were characterized by shorter height, lighter body weight, less experience in sports, and fewer training days per week.

Smaller body dimensions could be related to the ubiquitous pressure to be thin and lead to under-nutrition/under-fueling and, therefore, less muscle mass and poorer physical preparation. These could result in lower performance and/or enjoyment of sport. More athletes in leanness sports (e.g., pole vaulting, gymnastics) reported previously a BMI below 18.5 and eating disorders [10]. Moreover, significantly fewer ball game athletes are at risk of developing a female athlete triad (disordered eating, amenorrhea, and osteoporosis), having, at the same time, no underweight athletes [10]. There seems to be an inverse relationship between low BMI and a high number of athletes classified as at risk of developing eating disorders [46]. Therefore, athletes with lower body dimensions could be the ones requiring monitoring or prevention efforts.

Athletes with less experience in sports may be under more pressure to catch up with more developed peers, which may create prolonged stress. Similarly, training fewer days per week could be related to less engagement and possibly fewer opportunities to receive support from the sporting community. Unfortunately, these aspects have not been specifically researched and are, therefore, hard to compare.

### 4.6. Limitations

This study implemented a retrospective survey design, which is susceptible to sampling, response, and recall biases. However, as we asked for mental health complaints, these are possibly easier to remember due to their unpleasant symptoms.

All mental health complaints analyzed in this study were self-reported, although the question asked to list only the complaints diagnosed by a medical professional. However, our survey allowed for the athletes to provide honest and anonymous data. Therefore, the data we have gathered may be the closest representation of prevalence in this population.

The prevalence of mental health complaints was measured in this study by simply asking the athletes to report them; no validated tool was used. This method, however, allowed for us to measure the prevalence in the athletes who were aware of their suboptimal mental health and willing to report it anonymously and, therefore, was crucial to establishing the findings of this study.

## 5. Conclusions

Mental health complaints are present in collegiate athletes and are more prevalent in female than male athletes. Skill sports athletes reported the highest prevalence of mental health complaints within all the sports groups. Skill sports and female athletes are of special concern around well-being and mental health complaints and warrant targeting these groups for monitoring of early symptoms and implementation of preventive strategies. Anxiety and depression were the most reported and should be targeted in health prevention initiatives as the first ones. The self-reported prevalence found in this study is a magnitude lower than most of the studies available in the literature that mainly focused on the prevalence of mental health symptoms and not a diagnosis. In general, broader mental health education is needed for athletes, coaches, parents, and support personnel to improve reporting, lower the barriers to receiving consulting or treatment, and lower the stigma surrounding mental health in sports.

## Figures and Tables

**Table 1:** Athletes’ characteristics.

Characteristic	Athletes with Mental Health Complaints n = 269	Athletes with No Complaints n = 10,730	*p*-Value (Mann–Whitney or χ^2^)
Age, years; mean ± SD	19.9 ± 1.4	19.9 ± 1.3	0.650
Year at university; mean ± SD	2.2 ± 1.1	2.3 ± 1.2	0.440
Sex: female; n (%)	149 (3.6%/4096)	3947 (96.4%/4096)	**<0.001**
Sex: male; n (%)	116 (1.7%/6848)	6732 (98.3%/6848)	
Height, cm; mean ± SD	165.6 ± 9.0	168.3 ± 8.6	**<0.001**
Weight, kg; mean ± SD	61.6 ± 13.7	66.3 ± 14.1	**<0.001**
BMI; mean ± SD	22.3 ± 3.5	23.2 ± 3.6	**<0.001**
Sports experience, years; mean ± SD	8.0 ± 4.9	7.1 ± 5.1	**0.003**
Sports level in the past: national; n (%)	97 (36.1%)	4275 (39.8%)	0.554
Sports level in the past: international; n (%)	9 (3.3%)	322 (3.0%)	
Practice days per week; mean ± SD	4.8 ± 1.6	5.2 ± 1.3	**<0.001**
Reported at least one injury; n (%)	149 (55.6%/268)	5351 (49.9%/10,729)	0.064
Reported at least one illness; n (%)	45 (16.7%/269)	737 (6.9%/10,730)	**<0.001**
Support of athletic trainer; n (%)	138 (51.4%/269)	5811 (54.1%/10,730)	0.353

**Table 2:** Sports represented in this study divided into groups.

Skill Sports		Power Sports		Mixed Sports		Endurance Sports		Aesthetic Sports	
n = 592		n = 717		n = 8530		n = 958		n = 202	
5.4%		6.5%		77.6%		8.7%		1.8%	
Males n = 324		Males n = 478		Males n = 5332		Males n = 662		Males n = 52	
Females n = 262		Females n = 233		Females n = 3159		Females n = 293		Females n = 149	
Undisclosed n = 6		Undisclosed n = 6		Undisclosed n = 39		Undisclosed n = 3		Undisclosed n = 1	
Kendo	144	Judo	564	Lacrosse	1689	Athletics	531	Cheerleading	84
Aviation	84	Wrestling	49	Softball	1242	Rowing	195	Dance	35
Table tennis	83	Taekwondo	40	Baseball	1212	Swimming	123	Artistic swimming	17
Archery	58	Alpine skiing	22	American football	895	Orienteering	60	Figure skating	15
Kyudo	40	Shorinji kempo ※	15	Soccer	883	Canoeing	18	Gymnastics	12
Other (24 sports)	191	Other (6 sports)	27	Other (17 sports)	2609	Other (8 sports)	31	Other (5 sports)	39

※ Modern Japanese martial art based on Shaolin kung fu.

**Table 3:** Prevalence of mental health complaints in sports groups; n (%).

Complaint	Skill Sports	Power Sports	Mixed Sports	Endurance Sports	Aesthetic Sports	*p*-Value χ^2^	Total
Anxiety	11 (1.9)	3 (0.4)	77 (0.9)	10 (1.0)	1 (0.5)	**0.015**	102 (0.99)
Depression	10 (1.7)	5 (0.7)	65 (0.8)	9 (0.9)	5 (2.5)	0.084	94 (0.85)
Sleep disorders	9 (1.5)	5 (0.7)	57 (0.7)	5 (0.5)	4 (2.0)	**0.032**	80 (0.73)
Eating disorders	3 (0.5)	2 (0.3)	24 (0.3)	3 (0.5)	1 (0.5)	0.875	33 (0.30)
Developmental disorders	4 (0.7)	3 (0.4)	21 (0.2)	5 (0.5)	0 (0.0)	0.189	21 (0.19)
Alcohol misuse	0 (0.0)	0 (0.0)	3 (0.04)	0 (0.0)	1 (0.5)	**0.014**	4 (0.04)
Substance misuse	1 (0.2)	0 (0.0)	1 (0.01)	1 (0.1)	0 (0.0)	0.114	3 (0.03)
Total prevalence	24/592 (4.1)	11/717 (1.5)	198/8332 (2.3)	29/958 (3.0)	7/202 (3.5)	**0.020**	269/10,999 (2.4)

**Table 4:** Mental health complaints and disorders in females and males; n (%).

Complaint *	Females n = 4096	Males n = 6806	χ^2^ Pearson *p*-Value	Sex Unspecified
Anxiety	60 (1.5)	40 (0.6)	21 **<0.001**	2
Depression	51 (1.2)	42 (0.6)	12.142 **<0.001**	1
Sleep disorders	43 (1.0)	35 (0.5)	10.511 **0.001**	2
Eating disorders	27 (0.7)	5 (0.1)	30.206 **<0.001**	1
Developmental disorders	11 (0.3)	22 (0.3)	0.237 0.626	0
Alcohol misuse	1 (0.0)	3 (0.0)	0.264 0.607	1
Substance misuse	0 (0.0)	2 (0.0)	1.196 0.274	1
Total prevalence	149 (3.6)	116 (1.7)	40.984 **<0.001**	4

* Some athletes reported more than one complaint.

**Table 5:** Differences in characteristics between female athletes who reported mental health complaints versus those who did not.

Characteristic	Female Athletes Who Reported Mental Health Complaints n = 149	Female Athletes Who Did NOT Report Mental Health Complaints n = 3947	Mean Difference (95% CI) *	*p*-Value *t*-Test	Cohen’s d
Age; years	19.9 ± 1.3	19.8 ± 1.3	0.10 (−0.11–0.31)	0.340	0.08
Height; cm	160.2 ± 6.1	160.2 ± 5.8	0.00 (−0.19–0.19)	0.988	−0.01
Weight; kg	54.2 ± 7.6	55.5 ± 7.1	−0.06 (−1.07–0.94)	**0.031**	−0.18
BMI; m/kg^2^	21.1 ± 2.6	21.6 ± 2.3	−0.47 (−0.85–0.10)	**0.013**	−0.21
Sports experience; years	7.1 ± 5.4	7.9 ± 1.5	−0.82 (−1.61–0.15)	**0.046**	−0.17
Practice; days/week	4.8 ± 1.5	5.1 ± 1.3	−0.29 (−0.53–0.44)	**0.021**	−0.22

* 95% CI: 95% confidence interval; *p*-value < 0.05.

**Table 6:** Differences in characteristics between male athletes who reported mental health complaints versus those who did not.

Characteristic	Male Athletes Who Reported Mental Health Complaints n = 116	Male Athletes Who Did NOT Report Mental Health Complaints n = 6732	Mean Difference (95% CI) *	*p*-Value *t*-Test	Cohen’s d
Age; years	19.9 ± 1.6	19.9 ± 1.4	0.08 (−0.17–0.33)	0.541	0.06
Height; cm	172.5 ± 7.1	173.1 ± 5.9	−0.59 (−0.36–0.60)	0.291	−0.10
Weight; kg	71.0 ± 14.1	72.7 ± 13.3	−1.69 (−1.07–0.94)	0.173	−0.13
BMI; m/kg^2^	23.8 ± 3.9	24.2 ± 3.9	−0.44 (−0.85–0.10)	0.233	0.11
Sports experience; years	7.3 ± 5.0	8.1 ± 5.0	0.85 (−1.61–0.15)	0.078	−0.17
Practice; days/week	4.7 ± 1.7	5.2 ± 1.3	−0.47 (−0.53–0.4)	**<0.001**	−0.36

* 95% CI: 95% confidence interval; *p*-value < 0.05.

**Table 7:** Mean differences (95%CI) in each athlete characteristic between the athletes who reported mental complaint versus those who did not. Only statistically significant differences are presented.

Characteristic	Anxiety	Depression	Sleep Disorders	Eating Disorders	Developmental Disorders	Alcohol Misuse	Substance Misuse
Age; years	NS *	NS *	NS *	NS *	NS *	NS *	NS *
Height; cm	3.15 (1.48–4.82)	2.93 (1.19–4.67)	NS *	5.13 (22.19–0.19)	NS *	NS *	NS *
Weight; kg	4.45 (1.71–7.20)	4.27 (1.41–7.14)	NS *	8.37 (3.55–13.19)	NS *	NS *	NS *
BMI; m/kg^2^	NS *	NS*	NS *	1.70 (0.47–2.94)	NS *	NS *	NS *
Sports experience; years	NS *	1.02 (–0.05–2.05)	NS *	NS *	NS *	NS *	NS *
Practice; days/week	0.40 (0.15–0.65)	0.32 (0.06–0.58)	0.31 (0.03–0.60)	NS *	0.64 (0.19–1.08)	NS *	1.48 (0.02–2.95)

* NS—non-significant.

## Data Availability

The data are either presented in this manuscript or available from the corresponding author upon reasonable request due to privacy and ethical restrictions.
